# How the Innate Immune DNA Sensing cGAS-STING Pathway Is Involved in Apoptosis

**DOI:** 10.3390/ijms24033029

**Published:** 2023-02-03

**Authors:** Wanglong Zheng, Anjing Liu, Nengwen Xia, Nanhua Chen, François Meurens, Jianzhong Zhu

**Affiliations:** 1 College Veterinary Medicine, Yangzhou University, Yangzhou 225009, China; 2 Joint International Research Laboratory of Agriculture and Agri-Product Safety, Yangzhou University, Yangzhou 225009, China; 3 Comparative Medicine Research Institute, Yangzhou University, Yangzhou 225009, China; 4 Jiangsu Co-Innovation Center for Prevention and Control of Important Animal Infectious Diseases and Zoonoses, Yangzhou 225009, China; 5 BIOEPAR, INRAE, Oniris, 44307 Nantes, France; 6 Department of Veterinary Microbiology and Immunology, Western College of Veterinary Medicine, University of Saskatchewan, Saskatoon, SK S7N 5E2, Canada

**Keywords:** cGAS-STING, apoptosis, innate immunity, IFN, DNA sensing

## Abstract

The cGAS–STING signaling axis can be activated by cytosolic DNA, including both non-self DNA and self DNA. This axis is used by the innate immune system to monitor invading pathogens and/or damage. Increasing evidence has suggested that the cGAS-STING pathway not only facilitates inflammatory responses and the production of type I interferons (IFN), but also activates other cellular processes, such as apoptosis. Recently, many studies have focused on analyzing the mechanisms of apoptosis induced by the cGAS-STING pathway and their consequences. This review gives a detailed account of the interplay between the cGAS-STING pathway and apoptosis. The cGAS-STING pathway can induce apoptosis through ER stress, NLRP3, NF-κB, IRF3, and IFN signals. Conversely, apoptosis can feed back to regulate the cGAS-STING pathway, suppressing it via the activation of caspases or promoting it via mitochondrial DNA (mtDNA) release. Apoptosis mediated by the cGAS-STING pathway plays crucial roles in balancing innate immune responses, resisting infections, and limiting tumor growth.

## 1. Introduction

The innate immune system is a major contributor to the host defense in response to pathogen invasion and/or tissue damage. The initial sensing of infection and injury is mediated by pattern recognition receptors (PRRs), which recognize pathogen-associated molecular patterns (PAMPs) and damage-associated molecular patterns (DAMPs) [1]. DNA, as a PAMP or as a DAMP, can be sensed by PRRs to alert the cell about the presence of microbial pathogens as well as damaged or malignant cells in its vicinity, and triggers innate immune responses [2]. The cGAS-STING pathway is an important cytosolic DNA sensing pathway that can activate the expression of type I interferons (IFN) and inflammatory cytokines to induce an innate immune response and antimicrobial effects in response to non-self DNA and self DNA [3].

Besides regulating IFN production and inflammatory responses, accumulating evidence has shown that the cGAS-STING signaling pathway can also induce apoptosis after being activated by non-self DNA, self DNA, and some chemical agonists [4,5]. For instance, a study in vivo has shown that infection with herpes simplex virus type 1 (HSV-1) could cause brain immune cells to undergo cGAS-STING pathway-dependent apoptosis [4]. In addition, a study in vitro has shown that during human T cell leukemia virus type 1 (HTLV-1) infection, viral reverse transcription intermediates could cooperate with STING to promote the IRF3-Bax complex, needed for the subsequent apoptosis of human primary monocytes [6]. Further, a study on self DNA has suggested that DNA release from damaged nuclei and mitochondria caused by ultraviolet B could activate the transcription factor IRF3 and NF-κB pathway, leading to apoptosis though the cGAS-STING signaling pathway in human immortalized keratinocyte (HaCaT) cells [5]. Additionally, several agonists of STING can also induce an increase in apoptosis levels. The cGAS-STING pathway activation, by various STING agonists, including cyclic dinucleotides (CDNs) such as c-di-GMP, 3′3′-cGAMP, and 2′3′-cGAMP, and non-CDN such as lipophilic STING agonist diABZI, can also trigger the production of a large panel of proinflammatory cytokines and induce CD14^+^ monocyte apoptosis [7].

Cell death is the process by which cells are damaged and die. There are several types of cell death, including uncontrolled cell death (also known as necrosis) and programmed cell death. Necrosis is often considered as accidental death as it is generally seen as not controlled by the cell. Programmed cell death, different from necrosis, is an active cell death mediated by a cascade of gene expression events and can be mainly classified into apoptosis, autophagy, necroptosis, and pyroptosis [8]. Apoptosis is essential for many processes, such as the elimination of infected or transformed cells, the optimal activity of the immune system, the development of the organism, and normal cell turnover in the body to maintain homeostasis [9]. Apoptosis can take place either through extrinsic (death receptor-mediated) or intrinsic (mitochondrial) pathways. The intrinsic pathway is activated by a range of exogenous and endogenous stimuli, including DNA damage, ischemia, and oxidative stress, and is mediated by several members of the Bcl family. The Bcl family proteins bind to the mitochondrial membrane to regulate apoptosis [10]. The extrinsic pathway of apoptosis is initiated by a pro-death signal originating from outside the cell, most often triggered by natural killer (NK) lymphocytes or CD8-positive cytotoxic T lymphocytes (CTLs) [9]. However, the extrinsic pathway can crosstalk with the intrinsic apoptotic pathway via caspase-8-mediated cleavage of the BH3 interacting-domain death agonist (BID). The cleaved BID (known as the truncated form, tBID) triggers mitochondrial outer membrane permeabilization (MOMP) and cytochrome c release, thus initiating effector caspase activation and intrinsic apoptosis [11].

Considering that apoptosis exerts significant roles in regulating the immune response, many studies have been designed to explore the relation between apoptosis and the cGAS-STING pathway [12,13]. These studies provide data showing that apoptosis could promote the cGAS-STING pathway [12,14], whereas other research supports the idea that apoptosis could negatively regulate the cGAS-STING pathway [13,15]. Facing these complex and opposite conclusions, several questions naturally arise: How does the cGAS-STING pathway induce apoptosis? Why is the cGAS-STING pathway promoted or suppressed by apoptosis? What are the effects of cGAS-STING-mediated apoptosis during antiviral and antitumoral reactions? Thus, the aim of this narrative review is to summarize the available mechanisms and the consequences of cGAS-STING-activated apoptosis.

## 2. The Mechanisms of cGAS-STING Pathway-Induced Apoptosis

### 2.1. cGAS-STING Pathway Can Induce Apoptosis through Endoplasmic Reticulum Stress

**Figure 1:**
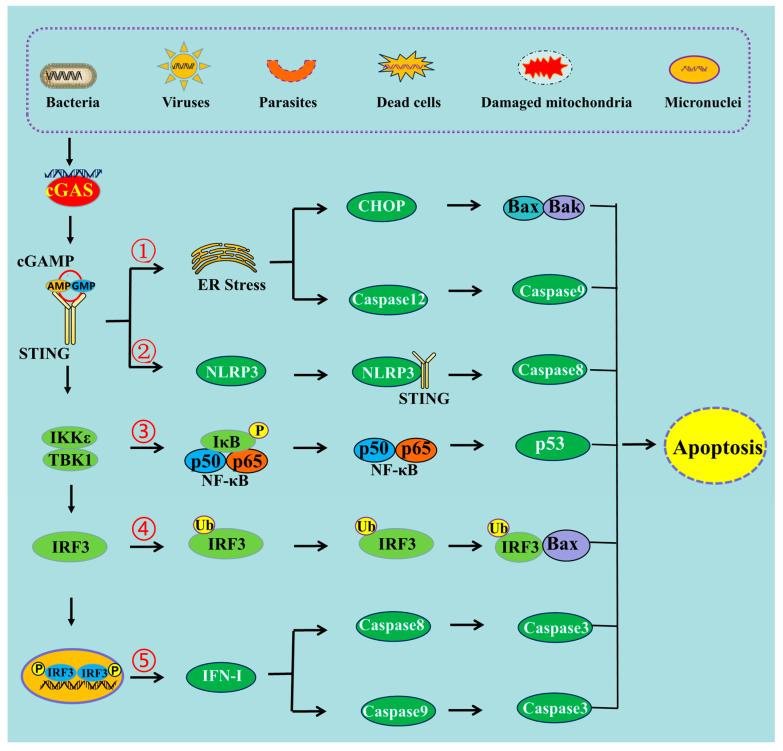
The schematic mechanism of cGAS-STING pathway-mediated apoptosis. ➀ cGAS-STING pathway can induce apoptosis through ER stress, ➁ through NLRP3 pathway, ➂ through NF-κB pathway, ➃ through the interaction of IRF3 and Bax, ➄ and through IFN-I production.

Studies have suggested that the activation of the cGAS-STING pathway could induce endoplasmic reticulum (ER) stress [16,17] (Figure 1). ER stress triggers a signaling reaction known as the unfolded protein response (UPR). The UPR is an adaptive cellular response that triggers reductions in protein synthesis and enhancements in ER protein-folding capacity and ER-associated protein degradation. If the adaptive response fails, cells are directed to undergo apoptosis [18]. This has indicated that co-transfecting plasmids of cGAS and STING into HEK293T cells, to transiently activate STING signaling, could upregulate the mRNA expression of ATF3 and GADD34, which are markers of ER stress and the UPR [19]. It has also been shown that STING is an important signal contributing to cardiac hypertrophy, and the expression levels of ER stress activation indicators such as p-PERK, p-IRE-1α, and p-eIF2α were observed to be stimulated after aortic banding surgery [20]. These ER stress activation indicators were markedly restrained in STING-KO mice [20]. Aortic banding surgery in mice is one of the most commonly used experimental models for cardiac pressure overload. Cardiac pressure overload is associated with high protein synthesis and Ca^2+^ dysregulation that could lead to ER stress [21].

It was observed that STING mediated ER stress and the UPR through a novel motif named “the UPR motif” [19]. Structural and functional analyses demonstrate that the UPR and the IFN response are mediated through distinct domains of STING [17]. The helix (amino acid—aa 322–343), and specifically residues R331 and R334, are critically needed for STING-mediated UPR. Indeed, deletion of the helix from the full-length STING (Δ322–343) can abrogate STING-mediated GADD34 induction [19]. Additionally, the cGAS-STING pathway may induce ER stress through an interaction between STING and Ca^2+^ sensor stromal interaction molecule 1 (STIM1) [22]. STIM1 and STING physically interact within the ER, primarily via their N-terminal domains. The physical and functional associations of STIM1 and STING are crucial for the maintenance of ER homeostasis [22]. STIM1 is known as a sensor of endoplasmic reticulum Ca^2+^ content, and has an essential role in the regulation of Ca^2+^ influx through specific plasma membrane store-operated Ca^2+^ channels [23].

An increasing number of studies suggest that the ER stress pathway plays an important role in cGAS-STING pathway-induced apoptosis [24]. ER stress can also induce apoptosis through various pathways, including the PERK and ATF6 pathways, the IRE1-TRAF2 pathway, and the caspase-12 pathway. Studies involving ischemia/reperfusion (I/R) experiments in rats have indicated that the activation of the cGAS-STING pathway could increase apoptosis through its regulation of ER stress, which causes pulmonary edema and pathological injury in the lungs. Then, the inhibition of the cGAS-STING pathway reduced the level of ER stress, attenuated injuries in the lung, and promoted pulmonary ventilation function in I/R rats [16]. Furthermore, Wu and collaborators have shown that STING is involved in the activation of ER stress and the UPR. This activation disrupts calcium homeostasis in T cells and makes T cells hyper-responsive to ER stress and the UPR, leading these cells to apoptosis [19]. Additionally, the cGAS-STING pathway mediates a crosstalk between ER stress and apoptosis during *Mycobacterium bovis* infection, enabling the control of this intracellular bacteria [25].

### 2.2. cGAS-STING Pathway Can Induce Apoptosis through NLRP3 Pathway

NOD-like receptor NLRP3 is a critical component of the innate immune system that mediates caspase-1 activation and the secretion of the proinflammatory cytokines IL-1β/IL-18 in response to microbial infection and cellular damage [26]. Stimulating STING not only triggers the activation of transcription factors IRF3 and NF-κB, but also prompts the activation of the NLRP3 inflammasome [27]. It has been proposed that the NLRP3 inflammasome is activated by the cGAS-STING pathway through the interaction between STING and NLRP3 [28]. STING binds NLRP3, and then it can promote the activation of the inflammasome via NLRP3 localization and the removal of NLRP3 polyubiquitination [28].

It was also observed that after infection with HSV-1 or stimulation by cytosolic DNA, both the K48- and K63-linked polyubiquitination of NLRP3 could be attenuated by STING [28]. The NLRP3 protein harbors several prototypic domains, including the PYRIN domain (PYD), NACHT-associated domain (NAD), and leucine-rich repeat (LRR) domain. Experiments showed that the domains of NAD and LRR were involved in the interaction with STING [28]. STING comprises five putative transmembrane (TM) regions. TM5 (151–160 aa, human) is involved in the interaction with NLRP3, while TM2 (41–81 aa, human) participates in the assembly and activation of the NLRP3 inflammasome [28]. Furthermore, another study has revealed that the cGAS-STING pathway could cause lysosomal damage and could induce a K^+^ efflux able to activate the NLRP3 inflammasome [29]. The monitoring of the intracellular level of K^+^ during DNA stimulation in BLaER1 monocytes showed a significant drop in intracellular K^+^ levels that was dependent on the cGAS-STING pathway [29].

Additionally, an increasing number of studies have shown that NLRP3 inflammasome activation contributes not only to pyroptosis but also to different types of cell death, including apoptosis [30]. Caspase-8 can act as a direct IL-1β-converting enzyme during NLRP3 inflammasome activation. Activated NLRP3-ASC inflammasomes recruit caspase-8 to drive IL-1β processing in murine bone marrow-derived dendritic cells (BMDC) independently of caspase-1 and caspase-11 [31]. If the inflammasome is activated but pyroptosis is blocked, caspase-8 can act as a backup and can drive cell death through the apoptotic pathway [32]. In the absence of caspase-1, NLRP3 inflammasomes directly utilize caspase-8 as both a pro-apoptotic initiator and a major IL-1β-converting protease [31]. Thus, it can be deduced that the cGAS-STING pathway can induce apoptosis through the activation of the NLRP3 pathway (Figure 1). A study carried out in mice demonstrated that STING induced inflammation and apoptosis in the heart by activating NLRP3. Indeed, STING knockout was able to inhibit the NLRP3-mediated inflammation and the apoptosis of cardiomyocytes [33].

### 2.3. cGAS-STING Pathway Can Induce Apoptosis through NF-κB

Nuclear factor κB (NF-κB) is one of the key regulators of inflammatory immune responses and is involved in the regulation of cytokine production [34]. It has been shown that the cGAS-STING pathway could drive NF-κB to induce the inflammatory response, and could mediate the immune response against pathogens [35,36]. The cGAS-STING pathway can activate NF-κB-dependent signaling transduction, thus regulating the transcription of genes encoding inflammatory cytokines [2]. After their activation by STING, TBK1 and its homolog IκB kinase epsilon (IKKε) activate the transcription factor NF-κB through the IKK complex [37]. STING, which is phosphorylated at Ser374 in humans (and Ser373 in mice), activates IKK during ER translocation. This event results in the phosphorylation of IκB and its ubiquitin–proteasome degradation, releasing the free NF-κB [38,39]. STING also activates the IKK complex on the Golgi apparatus and drives the free NF-κB into the nucleus [37].

Although NF-κB is well known for its anti-apoptotic function, it is also frequently reported that NF-κB activation exerts pro-apoptotic effects [40]. Regarding the anti-apoptotic effects of NF-κB, apoptosis is negatively regulated by NF-κB through the induction of multiple anti-apoptotic genes and occurs when NF-κB activation is compromised [41]. However, activation of NF-κB can also enhance the apoptosis induction of human osteosarcoma cells through the upregulation of the p53-upregulated modulator of apoptosis (PUMA) protein, also known as Bcl-2-binding component 3 (BBC3) [42]. Moreover, it has also been shown that NF-κB can cause apoptosis through the expression of pro-apoptotic genes [43].

STING can activate the NF-κB signaling cascade, whereas the blockage of NF-κB (using siRNA p65) signaling attenuates STING-induced apoptosis and senescence, and ameliorates STING-induced ECM metabolism imbalance [44]. Additionally, ultraviolet B (UVB) can induce apoptosis in human keratinocyte (HaCaT) cells through the activation of the cGAS-STING pathway [5]. Treatment with BAY, an inhibitor of the NF-κB pathway, can block UVB-induced apoptosis [5]. Thus, the NF-κB signal is involved in cGAS-STING pathway-induced apoptosis (Figure 1).

### 2.4. cGAS-STING Pathway Induces Apoptosis through IRF3-Bax Interaction

It has been shown that the proapoptotic role of the cGAS-STING pathway can be mediated by IRF3 (Figure 1), and that knocking down the expression of IRF3 can decrease the level of apoptosis [45]. Transcriptional profiling demonstrated that IRF3 initiated an antiviral response, but also rapidly induced cell death through the upregulation of a subset of proapoptotic genes [46]. It has also been recently identified that activated IRF3 could bind cytosolic Bax. This event results in mitochondrial outer membrane permeabilization (MOMP) and the release of cytochrome c. Then, cytochrome C damages the organelle and causes apoptotic cell death [47]. The strong interaction between IRF3 and Bax was proven by the co-immunoprecipitation and GST pulldown assay in cells infected with Sendai virus [48]. By contrast, several other members of the Bcl-2 family, including Bak, Bcl-xL, and Bcl-2, do not interact with IRF-3, indicating that the interaction is Bax-specific [48]. Indeed, IRF3 contains a Bcl-2 homology 3 (BH3)-like domain (G-[KQR]-[HKQNR]-[IV]-[KQR]), near its carboxyl terminus, which enables its interactions with Bax. BH3-like motifs (short peptide sequences) are commonly found among the members of the Bcl-2 family. To summarize, Bcl-2 related proteins control apoptosis through a complex network of protein–protein interactions mediated by BH3 domains [49].

The pro-apoptotic action of IRF3 could be distinct and independent of its transcriptional activity. From its N to C termini, the IRF3 protein contains a well-conserved DNA-binding domain (DBD), an IRF-associated domain (IAD) that facilitates dimerization, and an inhibitory domain (ID) that keeps IRFs in an inactive monomeric state until its activation by C-terminal phosphorylations. The C-terminal domain contains the critical serine residues (S385, S386, S396, and S398). The signal-dependent phosphorylation of these critical serine residues is required for activating IRF3 as a transcription factor [49]. Indeed, a mutant of IRF-3, missing S385, S386, S396, and S398, is unable to drive transcription, but can induce apoptosis. A drastic mutant of IRF-3, where the entire DBD was deleted and where the nuclear translocation and promoter-binding ability was missing, was also still able to mediate apoptosis, as assessed by PARP cleavage and caspase activation [49]. In order to explore the mechanism used by IRF3 to activate and mediate apoptosis, experiments were designed to analyze the role of ubiquitination in IRF3-induced apoptosis. It has been found that specific lysine residues of IRF3 are required to induce the apoptotic pathway, but not to induce the transcriptional pathway [50,51]. Among 14 lysine residues, lysine 193 and lysine 313 or 315 of human IRF3 and lysine 188 and lysine 306 or 308 of mouse IRF3 were necessary and sufficient to induce apoptosis [50].

### 2.5. cGAS-STING Pathway Induces Apoptosis through IFN-I Production

The cGAS-STING signaling pathway can sense viral infections and induce the production of type 1 interferons (IFNs) to combat the invading pathogens. Type 1 IFNs have been shown to induce apoptosis not only through the intrinsic pathway but also through the extrinsic pathway in various cell types [52,53]. For instance, IFN-β was shown to induce apoptosis through the intrinsic pathway via the down-regulation of PI3K/AKT signaling, the release of cytochrome c, and the activation of procaspase 9 in neuroblastoma cells [52]. However, another study showed that the induction of apoptosis by IFN-β is dependent on caspase-8 through the extrinsic pathway [54], and that apoptosis induced by IFN-β could be blocked using some inhibitors targeting caspase-8 but not caspase-9 [54]. Additionally, some studies have shown that IFN-I mediates apoptosis using the extrinsic signaling pathway. This process is also dependent on the expression of the death ligand TRAIL, in melanoma and breast cancer cells [55,56]. Therefore, the production of IFN-I may be implicated in the process of apoptosis induced by the cGAS-STING pathway (Figure 1).

Several signaling pathways are involved in the process of IFN-I regulating apoptosis. Among these pathways, the JAK-STAT and PI3K-AKT pathways appear to play crucial roles [52]. It has been suggested that IFN-I could induce apoptosis through the IFN-JAK-STAT pathway to regulate Bcl-2 family members [52]. IFN-I binds to the IFN-α/β receptors and then phosphorylates and activates two members of the JAK family: Tyk2 and JAK1. Then, these kinases further phosphorylate STAT1. The activation of STAT1 was found to be involved in the regulation of apoptosis. This was possible via the regulation of downstream Bcl-2 family members such as Bcl-2 and Bax through the ERK1/2 and JNK pathways [57]. Additionally, IFN-1 could also induce apoptosis through the suppression of the PI3K-AKT signaling pathway [52]. The PI3K/AKT signaling pathway is composed of serine/threonine protein kinases of the PI3K family, and plays an important role in the inhibition of apoptosis and the promotion of cell proliferation [58]. AKT can negatively regulate various pro-apoptotic BH3-only proteins at both the transcriptional and post-transcriptional levels through its effects on the transcription factors p53 and the Forkhead box protein O (Foxo) [59].

## 3. Does Apoptosis Promote or Inhibit the cGAS-STING Pathway?

### 3.1. Apoptosis Can Suppress the cGAS-STING Pathway through the Activation of Caspases

Caspases constitute a family of cysteine proteases, which are phylogenetically conserved throughout metazoans and have different roles [60]. According to their physiological functions, caspases could be classified into two categories: apoptotic caspases (caspases-2, -3, -6, -7, -8, -9, and -10 in mammals) and inflammatory caspases (caspases-1, -4, -5, and -12 in humans and caspases-1, -11, and -12 in mice) [32]. However, the usual classification of caspases as apoptotic or inflammatory has been strongly challenged in the last decade. Indeed, it became clear that inflammatory caspases such as caspase-1 can mediate cell death, including pyroptosis, whereas apoptotic caspases can mediate processes that are not linked to death [13]. During the apoptosis process, activated apoptotic caspases can regulate innate immune signaling and can prevent the dying cells from producing cytokines to keep the cells immunologically silent [15]. The apoptotic caspases, including caspase-9, -3, and -7, are essential negative regulators of the IFN-I production mediated by the mtDNA-induced cGAS-STING pathway [13]. In the absence of caspase-9, the cGAS-STING pathway is constitutively active and contributes to the IFN-dependent induction of ISG expression in mouse embryonic fibroblast (MEF) cells [13].

**Figure 2:**
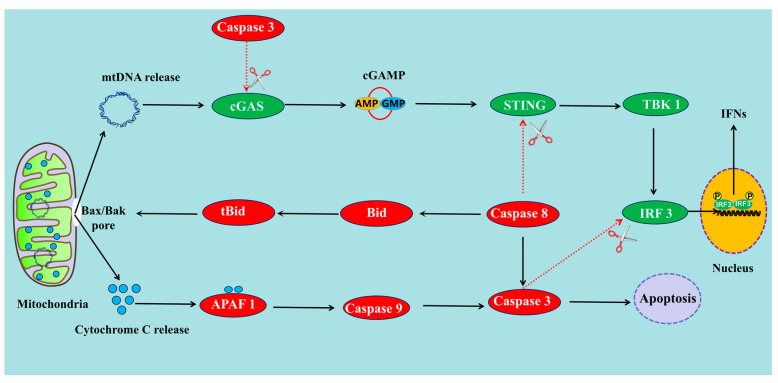
Apoptosis can feed back to regulate the cGAS-STING pathway. (1) Apoptosis can suppress the cGAS-STING pathway through the activation of caspases including caspase-3 and caspase-8. (2) Apoptosis can promote the cGAS-STING pathway through the release of mtDNA.

The cGAS-STING pathway can be inhibited by caspase-3 (Figure 2). Indeed, cGAS can be cleaved by apoptotic caspase-3 at position D319 during viral infection [15]. Human cGAS was found to have a typical caspase-3 recognition site, ISVD319, which is conserved among vertebrate cGAS proteins [15]. The D319 of cGAS is responsible for GTP binding and resides on the central β-sheet inside the cGAS catalytic pocket. Cleavage at position D319 results in the inactivation of cGAS catalytic activity and impairs cGAMP production [61]. IRF3 can also be cleaved by caspase-3 at position DD121/125 during apoptosis [62]. IRF3^−/−^ cells stably expressing IRF3-DD121/125AA showed enhanced IRF3 phosphorylation and ISG and IFN-I production, as well as decreased IRF3 cleavage, in response to different viruses or genomic DNA transfection [15]. An IFN-β luciferase report assay showed that the cleaved IRF3 fragments completely lost the ability to activate IFN-I promoter activity, while the DD121/125AA mutant induced even higher IFN promoter activity than the WT IRF3. IRF3 sequences from four different species (human, mouse, bovine, and pig) were aligned and three conserved aspartic acids, namely D116, D121, and D125, in human IRF3 were found [15]. The mutants DD121/125AA and DDD116/121/125AAA were completely resistant to ABT263 (Navitoclax), a Bcl-2 inhibitor, treatment-caused IRF3 cleavage [15].

Furthermore, the cGAS-STING pathway can be inhibited by caspase-8 (Figure 2). A study in *Bombyx mori* has shown that a caspase-8-like protein (Casp8L) was able to suppress the STING-mediated antiviral pathway [63]. The interaction between Casp8L and STING was identified by LC-MS/MS and immunofluorescence analysis. Overexpression of Casp8L can inhibit the cGAMP-STING-mediated antiviral signaling pathway [63]. Mice lacking caspase-8 in their keratinocytes develop cutaneous inflammation, which is not caused by TNF, IL-1, or TLR signaling, but rather by an increase in the transcription factor IRF3 in the epidermis [64]. Meanwhile, the keratinocytes lacking caspase-8 induce higher amounts of IFN-β and interferon-inducible proteins following transfection with double-stranded DNA than the wild-type keratinocytes [64]. Additionally, several inflammatory caspases, including caspase-1, caspase-4, caspase-5, and caspase-11, have also been identified as able to limit cGAS-mediated IFN-I production during DNA viral or bacterial infections [65].

### 3.2. Apoptosis Can Also Promote cGAS-STING Pathway through the Release of mtDNA

Mitochondrial DNA (mtDNA) is normally kept within the mitochondria, and can be released into the cytosol in response to stress [66]. When mtDNA leaks from damaged mitochondria into the cytosol, it can serve as a type of mitochondrial danger-associated molecular pattern (mtDAMP) and can engage various PRRs. The recognition of mtDAMPs by PRRs leads to the activation of the innate immune response (Figure 2). During apoptosis, mtDNA release is mediated by macro-pores in the mitochondrial outer membrane created by the oligomerization of the proteins Bax and Bak [66]. Specifically, Bax and Bak initiate MOMP by forming hetero- and homo-oligomeric pores in the outer mitochondrial membrane, so that Bax/Bak-mediated mitochondrial outer membrane pores gradually widen [67]. This allows the extrusion of the mitochondrial inner membrane into the cytosol, where the membrane is permeabilized, allowing mtDNA release into the cytosol.

mtDNA-dependent activation of cGAS-STING has been involved in a variety of pathophysiological processes, including infectious and inflammatory diseases [68]. Recently, it was suggested that mtDNA engages PRRs and triggers type I IFN and ISG expression in a cGAS-STING-dependent manner [68]. The mtDNA-dependent activation of cGAS-STING plays crucial roles in the defense against RNA and DNA viruses [69,70]. The cGAS-dependent signaling can be activated by the influenza A virus, a segmented negative-sense single-stranded RNA virus, via the release of mtDNA into the cytosol [71]. Furthermore, the induction of mtDNA release was also observed for *Herpesviridae*, double-stranded DNA viruses, to engage ISG expression [72]. Moreover, in addition to the induction of the IFN response, mtDNA has been reported to induce an inflammatory response via the activation of the cGAS-STING pathway in the adipose tissue, the liver, and the kidney [12]. Thus, the cellular monitoring of mtDNA homeostasis represents an additional sensory mechanism to robustly engage antiviral innate immunity [14].

## 4. What Are the Effects of the Apoptosis Process Induced by the cGAS-STING Pathway?

### 4.1. cGAS-STING-Mediated Apoptosis in Viral Pathogenesis, a Double-Edged Sword

Recently, accumulating evidence has shown that cGAS-STING-mediated apoptosis plays crucial roles in antiviral activity [73]. The IRF-3/Bax-mediated apoptotic signaling branch, activated by cytoplasmic DNA, contributes significantly to the host’s protection from viral infections and associated diseases [48]. The specific contribution of the cytoplasmic DNA-driven apoptotic pathway in viral replication was analyzed in Bax^−/−^ MEFs. Knocking out Bax from MEFs could elevate the replication of infectious Sendai virus [74]. Our previous study has shown that the porcine cGAS-STING pathway exerts an unusual antiviral function independently of the IFN and the autophagy process. In this situation, STING’s antiviral function may be mediated by apoptosis, or by several STING signaling events, including IFN, NF-κB, autophagy, and/or apoptosis, likely in a redundant manner [73].

However, other studies suggest that cGAS-STING-mediated apoptosis inhibits host antiviral activity [4]. Indeed, it has been shown that cGAS-STING-dependent apoptosis limits host antiviral activity in HSV-1-infected brain cells [4]. The blockage of caspase activity in brain cells was able to improve the clearance of HSV-1 and the outcome of infection [4]. It was observed that immune cells present at the sites of active HSV-1 replication were induced to produce IFN-I but underwent apoptosis when the immune-stimulatory signals became too strong. This mechanism may have evolved to preserve brain tissue from the damaging effect caused by the long-term activation of immune cells in the brain [4].

It is not surprising that the inhibition of cGAS-STING-mediated apoptosis has mixed effects on viral pathogenesis. As we have discussed above, cGAS-STING-mediated apoptosis can not only negatively regulate the cGAS-STING pathway but can also promote it. Apoptosis can suppress the cGAS-STING pathway through the activation of caspases and can promote the cGAS-STING pathway through the release of mtDNA. Furthermore, the timing of apoptosis is a critical factor in determining whether it will be pro- or antiviral. Indeed, in the early phase of infection, the host cells often use apoptosis to block viral spread, and apoptosis is therefore considered a potent antiviral defense mechanism by which infected cells are eliminated. In contrast, in the late phase of infection, apoptotic death of the infected cell may facilitate virus egress and the spread of the infection. Therefore, cGAS-STING-mediated apoptosis can be seen as a double-edged sword in viral pathogenesis.

### 4.2. The Effects of cGAS-STING-Mediated Apoptosis in Antitumor Immunity

The cGAS-STING pathway may also play an important role in antitumor immunity. It was indicated that the expression of cGAS and STING is usually inhibited in most cancer types, rather than being upregulated, especially when tumors are well-developed [75]. The signal of the cGAS-STING pathway was observed to be decreased in cervical cancer cells, and knocking down STING by siRNA enhanced the cell viability and the migration of cervical cancer cells. Conversely, the activation of STING inhibited the cell viability of cervical cancer cells [76]. Furthermore, after the transplantation of immunogenic tumors into syngeneic mice, tumors grew faster in STING-deficient mice than in wild-type mice [77]. Additionally, it has been demonstrated that STING agonists can also act in concert with a variety of anticancer therapies, leading to tumor regression [78]. STING agonists have promising biological activity and show excellent synergistic antitumor effects in combination with other cancer therapies such as radiotherapy, chemotherapy, or immune therapies, as has been proven in preclinical studies and some clinical trials [79]. Activation of STING by ADU-S100, a STING agonist, could inhibit cervical cancer tumor growth via the enhancement of the antitumor immune response [76].

Apoptosis is an important weapon of the immune system to eliminate damaged or abnormal cells and increasing evidence has indicated that the cGAS-STING pathway can eliminate cancer cells through the induction of apoptosis [80]. In cancer cells, the amount of cytoplasmic DNA is usually increased in response to the hostile milieu of the tumor micro-environment or the stresses caused by cancer therapeutics. Cancer cells with chromosomal abnormalities or genomic DNA damage often form micronucleus or cytoplasmic chromatin fragments, which can activate the cGAS-STING pathway to initiate a signaling cascade [81]. When the cGAS-STING pathway in tumor cells is activated, cytokines such as IFN-I are induced, leading to apoptosis [82]. Carboplatin, a second-generation platinum antitumor drug derived from cisplatin, can suppress human melanoma through the activation of cGAS-STING pathway-mediated apoptosis. Carboplatin activated the cGAS-STING pathway through the upregulation of TREX-1 expression in human melanoma [83].

## 5. Conclusions and Future Perspectives

The cGAS-STING pathway can induce apoptosis through ER stress, NLRP3, NF-κB, IRF3, and IFN signals. The apoptosis mediated by the cGAS-STING pathway is able to feed back and regulate the cGAS-STING signaling pathway. Apoptosis mediated by cGAS-STING plays crucial roles in balancing innate immune responses, resisting infections, and limiting tumor growth and transformation.

Damaged mitochondria trigger the innate immune response through the release of mtDNA to activate DNA-sensing pathways. This also initiates an immunologically silent response through activating caspases to cleave signaling molecules. However, during mitochondrial damage, the way in which the cell balances the immune system is still unclear. It is recommended that studies should be designed to explore this aspect in the future. Furthermore, the activated caspases not only mediate apoptosis, but also suppress the innate immune pathways through the cleavage of critical components. Viruses may employ the activated caspases to dampen the antiviral responses and facilitate their replication. Therefore, the impact of caspases should be considered during the investigation of the molecular mechanisms used by viruses to escape the innate immune system. Additionally, evidence has shown that the cGAS-STING pathway not only activates apoptosis but also induces other cellular processes, including autophagy, pyroptosis, and necroptosis. To confirm the relationships between apoptosis, autophagy, pyroptosis, and necroptosis induced by the cGAS–STING pathway, more attention should be paid in the fight against many pathological conditions.

## Data Availability

The data presented in this study are available in the article.
